# Review of Deep Learning Based Autosegmentation for Clinical Target Volume: Current Status and Future Directions

**DOI:** 10.1016/j.adro.2024.101470

**Published:** 2024-02-08

**Authors:** Thomas Matoska, Mira Patel, Hefei Liu, Sushil Beriwal

**Affiliations:** a Department of Radiation Oncology, Medical College of Wisconsin, Milwaukee, Wisconsin; b Department of Radiation Oncology, University of Pennsylvania, Philadelphia, Pennsylvania; c Varian Medical System Inc, Palo Alto, California; d Allegheny Health Network Cancer Institute, Pittsburgh, Pennsylvania

## Abstract

**Purpose:**

Manual contour work for radiation treatment planning takes significant time to ensure volumes are accurately delineated. The use of artificial intelligence with deep learning based autosegmentation (DLAS) models has made itself known in recent years to alleviate this workload. It is used for organs at risk contouring with significant consistency in performance and time saving. The purpose of this study was to evaluate the performance of present published data for DLAS of clinical target volume (CTV) contours, identify areas of improvement, and discuss future directions.

**Methods and Materials:**

A literature review was performed by using the key words “deep learning” AND (“segmentation” or “delineation”) AND “clinical target volume” in an indexed search into PubMed. A total of 154 articles based on the search criteria were reviewed. The review considered the DLAS model used, disease site, targets contoured, guidelines used, and the overall performance.

**Results:**

Of the 53 articles investigating DLAS of CTV, only 6 were published before 2020. Publications have increased in recent years, with 46 articles published between 2020 and 2023. The cervix (n = 19) and the prostate (n = 12) were studied most frequently. Most studies (n = 43) involved a single institution. Median sample size was 130 patients (range, 5-1052). The most common metrics used to measure DLAS performance were Dice similarity coefficient followed by Hausdorff distance. Dosimetric performance was seldom reported (n = 11). There was also variability in specific guidelines used (Radiation Therapy Oncology Group (RTOG), European Society for Therapeutic Radiology and Oncology (ESTRO), and others). DLAS models had good overall performance for contouring CTV volumes for multiple disease sites, with most studies showing Dice similarity coefficient values >0.7. DLAS models also delineated CTV volumes faster compared with manual contouring. However, some DLAS model contours still required at least minor edits, and future studies investigating DLAS of CTV volumes require improvement.

**Conclusions:**

DLAS demonstrates capability of completing CTV contour plans with increased efficiency and accuracy. However, most models are developed and validated by single institutions using guidelines followed by the developing institutions. Publications about DLAS of the CTV have increased in recent years. Future studies and DLAS models need to include larger data sets with different patient demographics, disease stages, validation in multi-institutional settings, and inclusion of dosimetric performance.

## Introduction

Radiation treatment planning involves a multistep, complex process requiring the use of CT simulations with manual segmentation of the gross tumor volume (GTV), clinical target volume (CTV), and organs at risk (OARs).^1^^,^^2^ Despite the expertise of radiation oncologists, manual segmentation remains time-consuming and can have large intraobserver and interobserver variability.^3^^,^^4^ The introduction of autosegmentation methods allow for uniformity and time efficiency. Early methods of autosegmentation included atlas-based methods using reference images with accompanied segmentation annotations to segment real-world clinical images. However, atlas-based autosegmentation has had limited clinical utility due to increased time required in building an atlas database, decreased algorithm performances when contour structures are altered or volumes are small, and large underdosages in the target volumes in comparison to deep learning based autosegmentation (DLAS).^5^, ^6^, ^7^ These limitations require more time editing contours by physicians. More recently, the implementation of DLAS has gained acceptance among radiation oncologists because of its superior performance and time savings.

DLAS involves artificial intelligence to perform autosegmentation on images by using a series of neural networks and architectures to analyze data.^8^ Convolutional neural networks (CNN) are a class of deep learning models that encompass deep learning artificial neural networks that make the assumption that inputs are images which can be used for contour outputs.^9^ CNNs have been frequently studied and assessed in DLAS studies. When assessing the performance of DLAS, metrics such as Hausdorff distance (HD) and Dice similarity coefficient (DSC) are considered. HD measures the average distance between ground truth image segmentation and autosegmentation or manual segmentation. A lower HD value indicates segmentation of higher quality.^10^ DSC compares the spatial overlap between 2 sets of contours.^11^ A DSC value ranges from 0 to 1, where a 0 indicates no spatial overlap between 2 sets of binary segmentation results, and a 1 indicates complete overlap. The greater the overlap, the better performance indicated by DLAS models. Specifically, a good overlap is considered to be DSC values greater than 0.700.^12^ Studies also commonly report subjective metrics regarding DLAS contours, including physician satisfaction and rating scales. Dosimetric outcomes are also another way to evaluate DLAS model contours. Extensive literature has been published on autosegmentation of OARs, which is more common in clinical practice today. Limited studies have investigated DLAS of the CTV, which includes expansions of the GTV volume to account for microscopic disease as well as prophylactic nodal regions.

Further investigation into the use of DLAS of the CTV is warranted to fully delineate its role in the future of radiation oncology. The goal of our review manuscript was to summarize and analyze the current literature reporting on the efficiency and performance of DLAS across different disease sites to delineate areas of strength and areas for improvement.

## Methods and Materials

**Figure 1:**
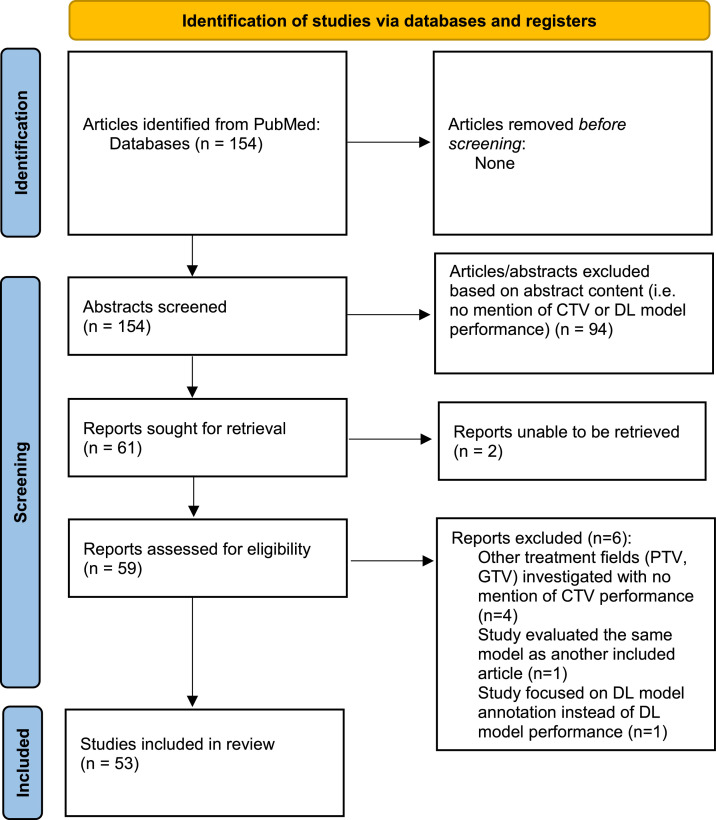
This figure displays the article screening and inclusion process utilized in this literature review.

A literature review was performed by using the key words “deep learning” AND (“segmentation” OR “delineation”) AND “clinical target volume” in indexed search into PubMed. A total of 154 articles based on the search criteria were reviewed. For this study the factors which were considered in our review were disease site, whether only the primary tumor was contoured or if lymph nodes were additionally included, the guidelines which were used, the model of deep learning based autosegmentation implemented, what type of imaging was used, whether the DLAS model being studied was developed in-house or commercially, whether dosimetric data were reported, and what the outcomes of CTV segmentation from the model were reported. Articles focusing on other methods of autosegmentation or the GTV and OARs without CTV were excluded. A PRISMA chart^13^ detailing article inclusion and exclusion methods is outlined in Fig. 1.

## Results

**Figure 2:**
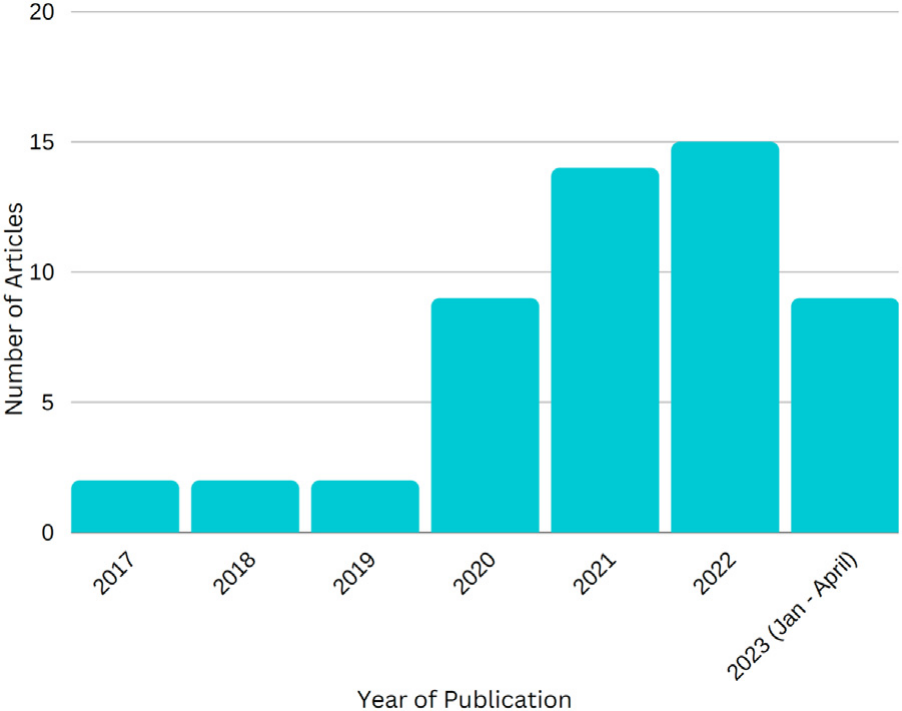
This figure displays the number of deep learning based autosegmentation of clinical target volume articles published each year from 2017 to April 2023.

There were 53 articles that met criteria for this review from 2017 to 2023. Of these, 47 articles were published articles from 2020 to April 2023 (Fig. 2). The cervix (n = 19) and the prostate (n = 12) were studied most frequently. Most studies (n = 43) involved a single institution compared with multi-institutional studies (n = 8). Median overall sample size was 130 patients (range, 5-1052). Common metrics used to measure DLAS performance were DSC followed by HD. A summary of all included articles from the literature review can be found in Table 1. A summary of disease site statistics can be found in Table 2. The results of each disease site are summarized below.

**Table 1:** The general characteristics and performance of DLAS model from each article included in this review

Study	Disease site	Guidelines	Lymph node	Model tested	Multi-institutional	Sample size	Geometric performance and outcome (CTV segmentation)	Time saving performance	Dosimetric performance
Sadeghi et al,^14^ 2023	Brain (glioblastoma, MRI)	Not specified	N	Segmentation net	Not specified	259	DL model achieved segmentation results with a DSC of 0.89 and HD of 1.49	Not reported	DL model had significantly lower Dmin CTV (minimum dose of CTV) and significantly higher Dmax (maximum dose of CTV) vs manual contouring
Bousabarah et al,^15^ 2020	Brain (metastases, MRI)	Not specified	No	Conventional U-Net (cU-Net) vs modified U-Net (mo-UNet) vs U-Net trained on small lesions	No	469	Quality of segmentations was >0.8 DSC for 52%, 0.6-0.8 DSC for 37% and <0.6 DSC for 11% of all detected lesions moU-Net and cU-Net performed similarly for DSC sU-Net performed worse among the 3 models Performance metrics were superior when all 3 models were combined	Not reported	Not reported
Choi et al,^18^ 2020	Breast (CT)	ESTRO and RTOG	Yes	FCDN vs atlas-based	No	62	FCDN had higher average DSCs for 11/14 CTV sites FCDN had lower average HD95 for 13/14 CTV sites	Not reported	Not reported
Men et al,^19^ 2018	Breast (CT)	Not specified	No	DDResNet vs DDNN, DDCNN	No	800	DDResNet had higher mean DSCs (0.91 in both breasts) and lower mean HD (10.5 R breast, 10.7 L breast) vs other models	Mean time for DDResNet CTV contouring 15 s vs 4 s (DDCNN) vs 21 s (DDNN) vs 10-20 min (manual)	Not reported
Zhong et al,^24^ 2023	Breast (CT)	Not specified	Yes	VB-Net vs manual	No	196	Model performed well based off DSC, HD, Jaccard index, for CTV volumes of chest wall, axillary nodes, supraclavicular nodes, but not for CTV internal mammary nodes	Not reported	Reported for OAR and planning target volume, no report on CTV
Chung et al,^20^ 2021	Breast (CT)	ESTRO and RTOG guidelines	Yes	2-stage CNN algorithm vs manual contour	No	111	CNN had mean DSCs >0.70 for all breast and regional lymph node CTVs Qualitative subjective scoring showed acceptable results for all CTV volumes	Not reported	Autosegmented contours had decreased dose coverage for axillary node levels I-III and internal mammary nodes
Almberg et al,^22^ 2022	Breast (CT)	ESTRO	Yes	Deep-learning model vs manual contouring	Yes (2)	200	Based on DSC and HD95, DL model outperformed manual for most structures Major corrections were required for 15% of CTVs	Not reported	D98 >95% dose coverage for DL model was fulfilled for 100% and 89% of the breast and LNs, respectively Dose coverage was poor in 3 cases related to internal mammary nodes
Buelens et al,^23^ 2022	Breast (CT)	International	Yes	3D CNN vs manual	Yes	95	CNN segmentation performance was best for breast CTV and worse for Rotter's space and the internal mammary nodes Guideline consistency improved from 77.14%-90.71% in favor of CNN segmentation	CNN segmentation saved on average 24 min per patient with a median time of 35 min for pure manual segmentation	Not reported
Dai et al,^21^ 2021	Breast (CT)	ESTRO	No	3D U-Net vs manual	Yes (2)	1052	Good DSC and HD95 scores were found for the most contours on pCT scan (DSC: 0.73-0.97, HD95: 2.22-9.36 mm) The mean DSC of CTV was 0.88 ± 0.03 for pCT, and 0.83 ± 0.03 for synthetic CT, respectively	Not reported	Mean ΔD90 and ΔD95 for CTV compared with reference were <2 and 4 Gy, respectively
Khalal et al,^16^ 2022	Breast (CT)	Not specified	No	U-Net vs ResUNet++ vs TernausNet	No	52	ResUNet++ performed best based on DSC, HD, and Jaccard coefficient All 3 models had DSC between 0.88 and 0.9	Not reported	Not reported
Liu et al,^17^ 2021	Breast (CT)	ESTRO and RTOG	No	U-ResNet (CNN) vs U-Net	No	160	U-ResNet had average DSC 0.94 and 95HD 4.31 mm, outperforming U-Net Vast majority (>99%) of CTV contours deemed acceptable for clinical treatment	Time for auto-segmentation of CTV and OARs was 10.03 s	Not reported
Ding et al,^30^ 2022	Cervix (CT)	RTOG	No	V-net vs U-net	No	130	Both V-Net and U-Net performed well for DSC, HD, JI, and average surface distance V‐Net CTV DSC (0.85) significantly higher compared with U-Net CTV (0.83)	Not reported	Not reported
Chang et al,^31^ 2021	Cervix (CT)	RTOG	Yes	3D ResU-Net	Yes (3)	400	Pretrained 3D ResUNet model that was trained with 50 additional cases had improved DSC and HD compared with the pretrained 3D ResUnet alone As additional test cases for model training increased, no significant difference in DSC whether evaluating a pretrained model vs model with no pretraining	Pretrained model saved more time with regards to DL model training compared with DL model with no pretraining	Not reported
Rigaud et al,^32^ 2021	Cervix (CT)	RTOG	No	2D-DeepLabV3+ vs 3-D 2-step U-Net vs manual	Yes (2)	247	2D model CTV: Mean DSC 0.84, mean HD 8.9 mm 3D model CTV: Mean DSC 0.85, mean HD 21.6 mm Interobserver variability (manual) primary CTV: Mean DSC 0.85, mean HD 12.2	Estimated time for DLAS segmentation with manual corrections <15 min	Dosimetric accuracy (V42.75 and D98) for 2D and 3D models lower than manually planned doses 2D model had higher V42.75 (98% and 100%) compared with 3D model (91% and 93%)
Ma et al,^33^ 2022	Cervix (CT)	RTOG, JCOG, FIGO	Yes	VB-Net vs manual	No	535	DL model accuracy was comparable with that of senior radiation oncologist (RO) and superior to that of junior/intermediate ROs Minor modifications were needed for 63.5% of auto-segmentations	Time savings for junior residents was 9.8 min for dCTV2 (parametrial area) and 28.9 for pCTV1 (pelvic LN CTV)	Not reported
Ma et al,^34^ 2022	Cervix (CT)	Not specified	Yes	3-channel adaptive auto-segmentation vs manual	No	107	TCAs with rigid registration of planning CT and TCAs with deformable registration of planning CT achieved superior DSC (0.89), MSD, and HD (6.14-6.28) compared with registration of planning CT alone	Not reported	Not reported
Chen et al,^44^ 2022	Cervix (CT)	RTOG	Yes	3D UNet vs manual	No	127	Not reported for CTV	Not reported	DL model had comparable percent coverage (>99%) of the CTV V42.75 and CTV V45 to manual contouring DL model had lower gamma passing rates for CTV (92.72%) vs manual contouring (98.77%)
Wang et al,^35^ 2020	Cervix (CT)	RTOG	Yes	DL model vs manual	No	125	DSC values of the auto‐segmentation model and manual contouring were 0.86 and 0.83 for the CTV, respectively Mean HD values for DL model (14.84 mm) were significantly better than manual contouring (18.37 mm) for the CTV	Auto‐segmentation mean delineation time for CTV and OARS: 2 vs 90 min for resident manual contouring	Not reported
Liu et al,^36^ 2020	Cervix (CT)	Consensus	Yes	DpnUNet	No	237	Mean DSC and 95HD values were 0.86 and 5.34 mm for the auto-segmented CTVs Clinical expert subjective assessment: 90% of the DL model contours were acceptable for clinical usage	Average DL model delineation time was within 15 s for both CTV and OARs	Not reported
Huang et al,^37^ 2023	Cervix (CT)	Consensus	Yes	MNet_IM vs other DL models	No	53	Most models performed well for CTV near the vagina, worse for CTV near cervix and uterus MNet_IM outperformed all other models for average surface distance, HD95, surface overlap, surface dice, volumetric dice	Not reported	Not reported
Xiao et al,^38^ 2022	Cervix (CT)	RTOG 0418	No	2D and 3D RefineNet vs other DL models	No	313	2DRefineNet and 3D RefineNetPlus3D had clinically acceptable CTV contours in postoperative cervical cancer patients DSC for RefineNet, FCN, U‐Net, context encoder‐Net, UNet3D, ResUNet3D, and RefineNet3D were 0.82, 0.80, 0.82, 0.81, 0.80, 0.81, and 0.82	Mean contour times of CTV ranged from 3.2-11.4 s	Not reported
Wang et al,^41^ 2022	Cervix (CT)	RTOG	Yes	Convolutional neural network vs manual	No	75	DL CTV auto-segmentation mean DSC: 0.77, 95HD: 5.81 mm, Jaccard coefficient: 0.62	Not reported	DL model had significantly lower V100 and Dmean (average radiation dose received by CTV) compared with manual contouring
Zhang et al,^25^ 2020	Cervix (brachytherapy) (CT)	Consensus	No	DSD-UNet vs 3D U-Net	No	91	DSD-UNet HR-CTV delineation DSC: 0.829, HD: 8.1 mm, Jaccard index: 0.72 DSD-UNet outperformed 3D-UNet	Time for segmentation of all structure volumes with DSD-UNet was 20 s	Not reported
Yoganathan et al,^29^ 2022	Cervix (brachytherapy) (MRI)	GEC Estro	No	Inception ResNet (2D and 2.5D) vs Resnet (2D and 2.5D)	No	39	2.5D models outperformed 2D models for intermediate and high-risk CTV Inception ResNet had better performance for HD and similar performance for DSC compared with ResNet	Time for contour predictions of DL models ranged from 25-45 s	D90 of HR CTV for manual contouring more similar to 2.5D models vs 2D models 2D models had significantly lower D90 compared with manual contouring
Hu et al,^16^ 2021	Cervix (brachytherapy) (CT)	Not specified	No	U-Net vs manual	No	70	U-Net contours had average DSC and HD95 0.89 and 1.66 mm, respectively Average tip and shaft errors of applicators were 0.80 and <0.50 mm compared with manual reconstruction	Mean segmentation time for DL model was 5.73 s	Dosimetric difference in D90 between manual and DL segmentation was 0.29%
Jiang et al,^27^ 2021	Cervix (brachytherapy) (CT)	GEC-ESTRO	No	RefineNet vs manual	No	200	RefineNet had higher DSC (0.861), HD (6.005 mm), and OI (0.839) than manually delineated CTV	Mean duration of DL contour of the CTV was 70 s	Not reported
Wang et al,^28^ 2023	Cervix (brachytherapy) (CT)	GEC-ESTRO	No	Convolutional neural network (CNN) vs manual	No	60	HR-CTV CNN DSC: 0.87, 95 HD: 1.45 mm, Jaccard coefficient: 0.78 80% of CNN HR-CTV contours needed no edits, 20% required minor edits	Not reported	Dose-volume indices (D90%, Dmean) for CNN vs manual for HR-CTV found no significant difference
Rayn et al,^43^ 2023	Cervix and prostate (nodes only) (CT)	Not specified	Yes	DL autosegmentation algorithm of Siemens Healthineers	Yes	103	DLAS contours rated on scale of 1 (requires complete recontouring) to 4 (requires minimal edits) 96% of female pelvic nodal region contours and 99% of male pelvic nodal region contours scored 3 or 4 No significant difference of any nodal region between males and females	Not reported	Not reported
Zabihollahy et al,^39^ 2022	Cervix (MRI)	Not specified	No	Superior-inferior CTV span is detected using Attention U-Net. CTV segmentation map is computed using 3D	No	125	DL model yielded mean DSC, mean absolute volume difference, and mean HD95 of 0.85, 13.47 cm^3^, and 3.70 mm	Average computation time including CTV segmentation is 41.23 s	Not reported
Shi et al,^40^ 2021	Cervix (CT)	RTOG	Yes	RA-CTVNet vs other DLAS models vs manual	No	462	RA-CTVNet performed better or comparably to 2 expert radiation oncologists RA-CTVNet outperformed all other DLAS models (DSC = 0.79) 3D-Unet had the worst performance out of all models (DSC = 0.688)	Not reported	Not reported
Cao et al,^48^ 2021	Esophagus (CT)	Not specified	Yes	Deep dilated convolutional U-Net (DDUNet) vs U-Net, U-Net with BN, attention U-Net	No	91	For CTV, DDUNet outperformed other 3 models with regards to DSC, 95HD, and Cohen kappa coefficient	Average time for DDUNet to contour CTVs was 25 s per patient	Not reported
Wong et al,^56^ 2021	H&N; prostate (CT)	Not specified	Yes	Deep learning model vs manual	Yes (2)	Sample Size H&N: 54 Sample size prostate: 93	H&N: Mean editing score (1-5, 5 = significant editing required) CTV neck LNs: 2 Mean satisfaction score (1-5, 5 = high satisfaction) CTV neck LNs: 4.8 Prostate: Mean editing score prostate: 2.8, mean editing score seminal vesicles: 1.7 Mean satisfaction score prostate + SV: 4.1 Mean DSC prostate: 0.88	Not reported	Not reported
Cardenas et all,^49^ 2021	H&N (CT)	Institutional	Yes	U-Net used to train 5 separate models	No	71	Mean DSC value: 0.816 When comparing the ensemble model results with each individual model's segmentations (ensemble - others), there was a mean (± SD) improvement of 0.01 ± 0.01 for the DSC Observed similar slight improvements in the mean surface distance and HD with ensemble approach	Mean time to contour all regions of interest was 6.0 min	Not reported
Men, Chen, Zhang et al,^50^ 2017	H&N (CT)	RTOG 0615	Yes	Deep deconvolutional neural network (DDNN) vs VGG-16	No	230	DDNN had higher average DSC (0.826% vs 0.737%) and lower HD (6.9 vs 11.1 mm) compared with VGG-16 DLAS CTV contours were close to manual contours with few corrections needed	Not reported	Not reported
Wong et al,^54^ 2020	H&N (CT)	Consensus	Yes (for H&N)	Deep learning model vs manual	No	20 per each disease site	Average DLAS DSC (0.72) significantly lower than manual DSC (0.79) for CTV	Time for DLAS contouring: 0.6 min; time for manual contouring: 26.6 min	Not reported
van der Veen et al,^57^ 2020	H&N (CT)	International consensus	Yes	CNN vs manual delineation	No	85	CNN delineations agreed very well with corrected delineation (all LN DSCs >0.7) DLAS performed best (DSC >85%) on LN levels Ib, II-IVa, VIa, VIb, VIIa, VIIb Interobserver variability was significantly smaller with CNN contours compared with manual delineations	Time taken for correcting CNN vs manual delineations were significantly shorter (35 vs 52 min)	Not reported
Weissmann et al,^55^ 2023	H&N (CT)	Consensus	Yes	DL model vs manual	No	55	Blinded expert rating for DL segmentations and manual were not significantly different Mean dice per level of DLAS 0.76 Adjustment of DL model to CT slice plane resulted in significantly better ratings compared with DL model without CT slice plane adjustment	DL autosegmentation lymph node level mean contouring time was 55.6 s	Not reported
Kihara et al,^52^ 2023	H&N (CT)	Not specified	Yes	3D U-Net with CT and GTV input vs U-Net CT input alone	No	310	U-Net with CT + GTV input had superior mean DSC (0.8) and average HD (3.0 mm) vs U-Net with CTV input alone (DSC: 0.76, average HD: 3.5 mm) For tonsillar cancer bilateral 1b LNs incorrectly delineated by U-Net CT and for base of tongue cancer ipsilateral 1b LNs incorrectly delineated by U-Net CT	Mean time for DL CTV delineation 0.86 s	Not reported
Xue et al,^51^ 2020	H&N (CT)	International guidelines	No	SI-Net vs U-Net	No	150	Average DSC and Jaccard index values from the SI-Net superior to U-Net for CTV (DSC: 0.84 vs 0.80, JI: 0.74 vs 0.69), SI-Net also had significantly lower average surface distance and HD compared with the U-Net (ASD: 2.8 vs 3.3, HD: 8.7 mm vs 9.7 mm)	Time for DL model CTV contours ranged from 13-20 s per patient compared with 10-20 min for manual contouring	Not reported
Cardenas et al,^53^ 2018	H&N (CT)	Not specified	Yes	DDN vs manual	No	52	DDN had median DSC 0.81, median mean surface distance 2.8 mm, median 95HD 7.55 for high-risk CTV volumes Patients with nodal disease had better agreement (DSC) between DDN and manual contour vs no nodal disease	DDN high risk CTV volumes mean delineation time 2.75 s	Not reported
Franssonet al,^58^ 2022	Prostate (MRI)	Not specified	No	2D U-Net vs deformable image registration algorithms	No	17	2D-UNet CTV autosegmentation DSC: 0.92, APL: 1642 DIR had significantly higher DSC and lower added path length (lower means less recontouring needed) compared 2D-UNet	Not reported	Not reported
Eppenhof et al,^59^ 2020	Prostate (MRI)	Not specified	No	U-Net vs Elastix (DIR algorithm)	No	5	Overlap loss U-Net and hybrid loss U-Net had significantly better DSC (0.86 and 0.86) and HD (5.82 mm and 5.66 mm) vs Elastix (DSC: 0.78, HD: 7.47 mm) Deformation loss U-Net had worse DSC and HD vs Elastix	Time to complete propagation of segmentation for all DL models was 0.49 s	Not reported
Balagopal et al,^61^ 2021	Prostate (CT)	RTOG	No	Physician style-aware network vs general model	No	456	DSC accuracy for all physicians was 3.4% higher on average than with a general model that does not differentiate physician styles A 5% DSC accuracy improvement was observed when adapting to the style of a separate institution DSC ranged from 0.756-0.892	Not reported	Not reported
Duan et al,^61^ 2022	Prostate (CT)	RTOG 0815	No	INTContour vs manual contour	No	107	INTContour demonstrated good performance of CTV contours with average DSC, HD95, and MSD were 0.83, 6.07 mm, and 2.07 mm, respectively 95.7% of INTContours were scored as either as “perfect” (34.8%) or “acceptable” (60.9%) in double-blinded evaluation	Not reported	Reported for OARs. Not reported for CTV
Hobbis et al,^62^ 2023	Prostate (CT)	FROGG	No	Custom DL model vs VT models	Not specified	Not specified	Custom DLAS model had median DSC 0.70, HD: 0.94 cm, and MSD 0.33 cm, outperforming VT models DLAS contours deemed acceptable by physicians in 54% of cases, vs 73% for manual contours	Mean time to edit DLAS contours that required major edits was 3 min and 49 s	Not reported
Balagopal et al,^63^ 2021	Prostate (CT)	Not specified	No	DL model	No	340	Overall, DL model performed well for CTV volumes based off DSC (0.87) and ASD (1.6 mm) U-Net outperformed PSPNet and DeepLab as backbone architecture DL models outperformed resident CTV segmentation 87% of the time	Not reported	Not reported
Min et al,^64^ 2021	Prostate (MRI)	Not specified	No	UNet with anatomic gate in an AtlasNet (AN-AG Unet), UNet, multichannel UNet within Atlast Net (AN M-UNet), multichannel UNet (M-UNet), 3D UNet, SG-MA	Yes (5)	393	AN-AG UNet performed best for CTV delineation (DSC: 0.9) AN-AG UNet achieved the highest AUC for delineation QA classification CTV DSC >0.7 for all models except UNet	Not reported	Not reported
Shen et al,^65^ 2023	Prostate (CT)	ESTRO ACROP	No	CUNet vs manual	No	217	DL CTV auto-segmentation mean DSC: 0.84, mean 95HD: 5.04 mm Blind physician evaluation resulted in selection of DL auto segmentation more often than manual contouring	Avg delineation time for DL auto-segmentation: <15 s	Not reported for CTV. Only reported for PTV
Kawula et al,^66^ 2022	Prostate (CT)	Not specified	No	3D-UNet vs manual	No	69	DL prostate auto-segmentation DSC: 0.87, mean HD: 1.6 mm	Not reported	DL surrogate CTV showed agreement with manual contours regarding D98%, D2%, V95%. These were within 2% and 3 Gy of reference except for one case
Elguindi et al,^67^ 2019	Prostate (MRI)	Not specified	No	DeepLabV3+ vs U-Net	No	50	DeepLabV3+ had average volumetric DSC 0.83 and surface DSC 0.85 vs compared with U-Net	Not reported	Not reported
Men et al,^45^ 2017	Rectum (CT)	Not specified	Yes	DDCNN vs U-Net	No	278	DDCNN outperformed U-Net with higher average DSC (0.87 vs 0.81)	Mean time for model auto-segmentation for target + OARs was 45 s	Not reported
Wu et al,^46^ 2022	Rectum (CT)	RTOG	Yes	U-Net vs manual	No	135	U-Net CTV DSC: 0.9, 95HD: 8.11 mm U-Net had better performance compared with manual based on blinded clinician scoring	Avg time to U-Net segmentation was 15 s vs 45-60 min for manual	Not reported
Song, et al,^47^ 2020	Rectum (CT)	International and institutional-specific	Yes	DeepLabv3+ vs ResUNet	No	209	DeepLabv3+ had higher mean volumetric (0.88 vs 0.87) and surface DSC (0.79 vs 0.78) for CTV vs ResUNet	Mean DeepLabv3+ and ResUNet computing time for CTV + OARs were 27.83 and 34.66 s, respectively Mean manual correction time for CTV DeepLabv3+ and ResUNet 11.17 and 7.29 min, respectively	Not reported

*Abbreviations:* 3D = 3-dimensional; APL = added path length; ASD = average surface distance; BN = batch normalization; CNN = convolutional neural network; CT = computed tomography; CTV = clinical target volume; DDCNN = deep dilated convolutional neural network; DDN = deep neural network; DDResNet = deep dilated residual network; DL = deep learning; DLAS = deep learning based autosegmentation; ESTRO = European Society for Therapeutic Radiology and Oncology; FCDN = fully convolutional DenseNet; H&N = head and neck; HR = high-risk; JI = Jaccard index; LN = lymph node; MRI = magnetic resonance imaging; OAR = organs at risk; OI = Overlap Index; pCT = planning computed tomography; RTOG = Radiation Therapy Oncology Group; SG-MA = structure guided multiatlas; TCA = three channel adaptive autosegmentation; VT = vendor trained.

**Table 2:** Summary of DLAS studies across disease site, including number of articles, sample size, surgical status, in-house model use, and performance metric breakdown and summary

Disease site	No. of articles	Median sample size to train (range)	Median sample size to validate / evaluate performance (range)	Breakdown by radiation setting (postsurgery, brachytherapy, etc)	In-house model vs commercial model	Clinical performance metrics utilized	Primary tumor or postsurgical bed mean CTV DSC and HD range	Lymph node CTV DSC and HD range
Brain	2	382.5 (296-469)	27.5 (15-40)	Glioblastoma (n = 1) Brain metastases (n = 1)	All in-house models	DSC (n = 2), Concordance correlation coefficient (n = 1), HD (n = 1), dosimetry data (n = 1)	CTV DSC: 0.7-0.89 HD (GBM): 1.49 mm	None reported
Breast	9	128 (35-700)	33.5 (19-352)	Postlumpectomy (n = 7) Postmastectomy (n = 2) Postchemotherapy (n = 1)	All in-house models	DSC (n = 9), HD (n = 9), qualitative or subjective (n = 4), dosimetry (n = 3), Jaccard index (n = 2), Mean surface distance (n = 1), relative volumetric distance (n = 1)	CTV whole breast DSC: 0.83-0.95 HD: 3.7-19.57 mm CTV chest wall, DSC: 0.73-0.736 HD: 16.3-29.3 mm	Axillary (level 1-3) - DSC: 0.726-0.79 HD: 4.15-15.7 mm Internal mammary - DSC: 0.51-0.75 HD: 3.3-45.41 mm Supraclavicular - DSC: 0.72-0.78 HD: 5.78-19 mm Rotter's space, DSC: 0.637 HD: 6.8 mm
Cervix (brachytherapy)	5	61.5 (40-160)	20 (19-50)	Postexternal beam radiation (n = 3), postexternal beam radiation, not specified (n = 2)	All in house models	DSC (n = 5), HD (n = 5), dosimetry (n = 3), Jaccard index (n = 2), qualitative or subjective (n = 1), overlap index (n = 1)	CTV DSC: 0.71-0.89 HD: 1.45-11.03 mm	None reported
Cervix (external beam)	14	134.5 (10-300)	37.5 (13-81)	No surgery (n = 6), both no surgery patients and postoperative (n = 4), postoperative (n = 1), not specified (n = 1)	In-house (n = 13), commercial (n = 1)	DSC (n = 11), HD (n = 10), average surface distance (n = 4), Jaccard index (n = 3), dosimetry n = 3), qualitative or subjective (n = 2), mean surface distance (= 2), absolute volume difference (n = 1), surface and volumetric dice (n = 1), distance to agreement (n = 1), interobserver variability (n = 1)	CTV DSC: 0.68-0.89 CTV HD: 3.2-21.6 mm	Some nodal CTVs were included with primary CTV Following data from node only (n = 1, Ma et al) CTV DSC: 0.86-0.88 CTV HD: 20.78-21.6 mm
Esophagus	1	58	33	Postesophagectomy	In-house	DSC, HD, Cohen kappa coefficient	CTV DSC: 0.835-0.867 HD: 19.4-23.6 mm	None reported
Head and neck	9	102 (35-250)	20 (15-60)	No surgery (n = 6), not specified (n = 2), included no surgery and postoperative patients (n = 1)	In-house (n = 7), commercial (n = 2)	DSC (n = 6), HD (n = 5), mean surface distance (n = 3), qualitative or subjective (n = 3), interobserver variability (n = 1), volumetric and surface dice (n = 1), Jaccard index (n = 1), average surface distance (n = 1)	CTV (primary ± nodes) DSC: 0.72-0.84 HD: 3.5- 11.1 mm	Some nodal CTVs were included with primary CTV Following data from node only (n = 2) studies: Cardenas et al^47^ and van der Veen et al^57^ CTV DSC (combined for all nodal levels, I-VI): 0.819-0.897 CTV HD (combined for all nodal levels, I-VI, van der Veen et al^57^): 7.9 mm
Prostate	12	72 (17-313)	28 (5-143)	No surgery (n = 9), postoperative (n = 3)	In-house (n = 8), Commercial (n = 4)	DSC (n = 10), HD (n = 6), qualitative or subjective (n = 6), mean or average surface distance (n = 4), added path length (n = 1), volumetric and surface dice (n = 1), dosimetry. (n = 1)	CTV DSC: 0.65-0.92 HD: 0.94-6.29	None reported
Rectum	3	122 (98-218)	60 (13-111)	Neoadjuvant setting (n = 2), postoperative (n = 1)	All in-house models (n = 3)	DSC (n = 3), qualitative or subjective (n = 2), HD (n = 1)	CTV DSC: 0.78-0.9 HD (n = 1): 8.11 mm	Nodes included in primary CTV

*Abbreviations:* CTV = clinical target volume; DLAS = deep learning based autosegmentation; DSC = Dice similarity coefficient; GBM = Glioblastoma Multiforme; HD = Hausdorff distance.

### Brain

The contouring of CTV for high-grade gliomas (n = 1) and brain metastases (n = 1) has been explored with DLAS without multi-institutional data. Both studies used in-house models and magnetic resonance imaging (MRI) scans for DLAS contouring of CTV volumes. Sample size to train and sample size to validate ranged from 296 to 469 patients and 15 to 40 patients, respectively. DSC was used to measure clinical performance of DLAS model in both articles. Sadeghi et al described a modified Segmentation-Net (SegNet) model that achieved a mean DSC of 0.896 and mean HD of 1.49 mm in patients with unresected glioblastoma. This model also revealed a statistically significant difference between D_min_ and D_max_ for automatically delineated CTV versus manual contours; however, no differences were found between D_mean_ and D_98%_ of the CTV for both sets of contours.^14^ In regard to brain metastases, there was one report of agreement between the manually and automatically assess tumor volumes quantified by a concordance correlation coefficient of 0.87, and a mean DSC for brain metastases to be 0.7 for a NetSUM model combining multiple individual DLAS models through a summation technique.^15^ Overall, the performance of DLAS in CNS malignancies, compared with manual segmentation, are clinically acceptable. More literature regarding DLAS in all CNS malignancies, including glioblastoma, meningioma, and brain metastases, is required.

### Breast

DLAS of CTV volumes after breast conserving surgery (n = 7) was studied most frequently followed by CTV volumes after mastectomy (n = 2) and after chemotherapy without surgery (n = 1). Studies used ESTRO, RTOG, or some other international guidelines and more than half (n = 5, 56%) of the studies included CTV lymph nodes. Most studies were performed and validated by a single institution or 2 institutions. All breast cancer studies used in-house DLAS models and CT scans for model training and contour delineation. Median sample size to train and validate/test performance of DLAS models were 128 patients (range, 35-700) and 33.5 patients (range, 19-352), respectively. DSC (n = 9), HD (n = 9), and qualitative rating measures (n = 4) were commonly used to assess performance.

Subjective rating performance was good and mean DSCs for DLAS models were ≥0.7 for whole breast postlumpectomy or postchemotherapy (DSC range, 0.83-0.95) and chest wall postmastectomy (DSC, 0.73-0.736). Performance was comparable between CTV right and left breast in studies that reported CTV performance in both breasts.^16^, ^17^, ^18^, ^19^, ^20^, ^21^, ^22^, ^23^, ^24^ Dai et al performed a multi-institutional study that reported better performance for DLAS models on planning CT scans compared with scanning CT scans.^21^ Lymph node CTVs are the most difficult fields for DLAS models. Studies reported worse DLAS performance for CTV internal mammary lymph nodes (DSC, 0.51-0.60) and Rotter's space (DSC, 0.63).^23^^,^^24^ Choi et al reported on several different lymph node levels with the lowest DLAS model performance for ESTRO guideline left CTV supraclavicular nodes (DSC, 0.7).^18^ Almberg et al reported worst DLAS model performance for CTV interpectoral lymph nodes (DSC, 0.7).^22^ The DLAS model from Chung et al had a mean CTV level 3 axillary lymph nodes of 0.64. This model had poor performance for ESTRO guideline supraclavicular lymph nodes (DSC, 0.67) and intramammary nodes (DSC, 0.67) with CT scans with contrast.^20^ Mean contour time for one DLAS model was between 4 to 21 seconds.^19^ Buelens et al reported an average of 11 minutes saved per patient with DLAS versus. manual segmentation.^23^ Of the articles that reported dosimetric data, the differences among autosegmented and manual contours were minimal. However, articles revealed autosegmented contours had decreased dose coverage for axillary node levels I to III and internal mammary nodes.^20^ Mean ΔD_90_/ΔD_95_ for autosegmented CTV was less than 2/4 Gy compared with original manual contour plans.^21^ Overall, DLAS of the breast is efficient and effective for CTV whole breast and CTV, but DLAS of certain draining lymph nodes needs improvement.

### Cervix (brachytherapy)

DLAS of brachytherapy CTV for cervical cancer have been studied after external beam radiation (n = 3), and other studies (n = 2) did not specify the treatment given before brachytherapy (n = 2). GEC-ESTRO were commonly used. All 5 studies were performed by a single institution and used in-house DLAS models. One study used MRI (vs 4 studies using CT imaging). Median sample size to train and validate/test performance of DLAS models were 61.5 (R, 40-160) and 20 (R, 19-50), respectively. DSC (n = 5), HD (n = 5), and Jaccard index (n = 2) were used most frequently to assess model performance.

Overall, DLAS models performed well for cervical brachytherapy CTV volumes using CT imaging with DSC ranges between 0.83 and 0.89.^25^, ^26^, ^27^, ^28^ Zhang et al compared 2 DLAS models, a novel 3-dimensional (3D) CNN to the standard 3D U-Net, in which the proposed novel model outperformed the standard model and was deemed by physicians to improve efficiency and consistency of treatment planning.^25^ In another study comparing a proposed BT DLAS with manually defined contours, DLAS contours evaluated by physicians were shown to be satisfactory without edits.^28^ Yoganathan et al used 2D and 2.5D ResNet and Inception ResNet models with MRI imaging, showing worse performance of 2D models compared with 2.5D models.^29^ These models also had worse performance for intermediate risk CTV volumes (DSC, 0.71-0.75). Regarding time savings, Jiang et al reported their DLAS model cut down 60% of total time compared with manual delineations with a mean duration to contour CTV of 70 seconds.^27^ It was also more time efficient, cutting down 60% of total time compared with manual delineations. From the articles that reported dosimetric data, certain autosegmentation models performed better than others. For example, in comparison to the 2-dimensional (2D) model, which had significantly lower D_90_ values compared with manual contours, the D_90_ of CTV for manual contours was similar to 2.5D models.^29^ Other models had minimal to no significant dosimetric differences between manual and autosegmented contours.^26^^,^^28^ Overall, DLAS of brachytherapy CTV for cervical cancer is efficient and accurate, but more studies with MRI imaging and in postoperative settings are warranted.

### Cervix (external beam) 13 CT 1 MRI

DLAS of cervical CTV volumes with external beam radiation has been studied most in patients with no surgery (n = 6). RTOG guidelines were most commonly used among studies. Only 3 included studies were performed or validated by multiple institutions. CT imaging (n = 13, vs 1 MRI study) and in house DLAS models (n = 13) were commonly used. Median sample size to train and validate DLAS models was 134.5 (R, 10-300) and 37.5 (R, 13-81), respectively. DSC (n = 11) and HD (n = 10) were used most frequently to assess model performance of cervical CTV volumes

DLAS models performed well in contouring cervical cancer CTV, with a DSC range of 0.68 to 0.89.^30^, ^31^, ^32^, ^33^, ^34^, ^35^, ^36^, ^37^, ^38^, ^39^, ^40^, ^41^ Only Chang et al showed one pretrained DLAS model that had DSC of 0.68.^31^ Compared with manual contouring, DLAS models used demonstrated satisfactory, if not better, performance than manual contouring with improvements in DSC and HD.^26^^,^^27^^,^^30^^,^^32^, ^33^, ^34^, ^35^, ^36^, ^37^, ^38^^,^^40^^,^^42^ When looking at subjective performance metrics, DLAS model accuracy was comparable with that of senior radiation oncologists and superior to that of junior and intermediate radiation oncologists.^34^ Rayn et al evaluated DLAS of pelvic lymph node volumes across multiple institutions, reporting 96% of contours requiring a few or minimal edits.^43^ When evaluating CTV coverage, Chen et al reported 99.86% coverage of the CTV V42.5 and 99.47% coverage of the CTV V45 for the DLAS model.^44^ When reporting time savings, one study reported an estimated time for DLAS contouring with manual corrections to be <15 minutes.^32^ Other studies found time savings of 88 minutes when comparing DLAS versus resident contouring and 9.8 to 28.9 minutes saved for junior residents when contouring cervical nodal or parametrial volumes.^34^^,^^35^ When considering dosimetric data, certain models had lower dosimetric accuracy regarding V_42.75_, V_100_, and D_mean_. Specifically, although the 2D model had a higher V_42.75_ compared with the 3D model, both models were lower in accuracy in comparison to manual contours.^32^ However, one article reported comparable percent coverage of CTV V_42.75_ and V_45_ for the DLAS model to the manual contours.^44^ Overall, the use of DLAS revealed improvement in accuracy of CTV contours for the cervix with accompanied time savings for both more senior radiation oncologists and residents; however, more emphasis and improvement on dosimetric performance of DLAS models is required.

### Gastrointestinal (rectum and esophagus)

Current literature for DLAS of CTV in gastrointestinal malignancies focuses on neoadjuvant setting for rectal cancer (n = 2) and postoperative settings in both rectal (n = 1) and esophageal cancer (n = 1) at single institutions using RTOG or other international and institutional guidelines. CTV volumes for rectal cancer all included regional lymph nodes. All gastrointestinal studies used in-house models and CT scans for model training and CTV delineation. Median sample size to train models was 110 patients (R, 58-218) and median sample size to validate/test models was 46.5 (R, 13-111). Common performance metrics used were DSC (n = 4), qualitative or subjective metrics (n = 2), and HD (n = 2).

DLAS model performance of CTV for rectal cancer was good for studies investigating preoperative and postoperative radiation therapy, with mean CTV DSCs ranging from 0.78 to 0.9.^45^, ^46^, ^47^ Wu et al found DLAS model had better performance based on a blinded subjective scoring system compared with manual contouring. DLAS models were also more efficient than manual contouring. The range for mean time for DLAS contour creation was 15 to 45 seconds for CTV and OARs. Song et al reported mean CTV correction time for 2 DLAS models to be 7.29 and 11.17 minutes.^47^ Cao et al investigated a 5-fold cross validated DLAS model to segment CTV lymph nodes and CTV esophageal tumor bed after an esophagectomy. For various DLAS models in this study, DSCs range was 0.83.5 to 0.867. Average time to perform CTV contour for one DLAS model was 25 seconds.^48^ DLAS models efficiently and accurately contoured CTV of rectum and esophagus. However, more studies investigating radiation in both neoadjuvant and adjuvant surgical settings for rectal cancer and neoadjuvant settings for esophageal cancer are required before widespread clinical implementation.

### Head and neck

For studies investigating DLAS of head and neck cancers, most studies (n = 6) were in upfront radiation settings without surgery. One study included patients with no surgery and patients in postoperative setting. All studies were performed by a single institution and used CT scans for treatment planning. Most DLAS models were in-house (n = 7) and other models were commercial (n = 2). Commonly used guidelines included RTOG or international guidelines. Nodes were included in 8 out of 9 studies with 2 studies reporting on lymph nodes only. Median sample size to train and validate/test DLAS models were 72 (R17-313) and 28 (5-143) patients, respectively. DSC (n = 6) and HD (n = 5) were most used to assess DLAS model performance.

Several studies noted well performing models for CTV primary ± CTV lymph nodes based off DSCs (range, 0.72-0.84) or good subjective performance scores comparable with manual contouring. Generally, few edits of the DLAS model contours were required.^49^, ^50^, ^51^, ^52^, ^53^, ^54^, ^55^, ^56^, ^57^ Wong et al found the commercial DLAS model that was used had worse performance (DSC, 0.72) compared with manual contouring, although the model led to fast contouring of CTV volumes. More data are needed to compare the performance of commercial and in-house DLAS models. Some studies reported data specific to head and neck lymph nodes. Cardenas et al reported better DSC performance in patients with lymph node involvement compared with those without lymph node involvement.^53^ Weissman et al reported improved DLAS performance when the model was adjusted to the CT slice plane compared with when the model was not adjusted to the CT slice plane.^55^ van der Veen et al reported best DLAS performance for LN levels Ib, II-IVa, VIa, VIb, VIIa, and VIIb (DSC, 0.85), and Kihara et al reported their DLAS model incorrectly segmented 1b lymph node levels for tonsillar and base of tongue cancer.^52^^,^^57^ van der Veen et al also measured time to DLAS of all lymph node levels to be 86 seconds with the time needed to correct autosegmented contours of lymph nodes (35 minutes) to be less than time to correct manual contours (52 minutes).^57^ Reported mean times to delineate CTV ranged from 0.86 to 20 seconds.^51^^,^^52^ Overall, DLAS models are successful in efficiently contouring CTV volumes similar to ground truth contours for head and neck cancer, although the development and validation of these models are limited to a single institution.

### Prostate

CTV volumes for DLAS of prostate most frequently included a combination of the prostate ± seminal vesicles (n = 9), followed by postsurgical bed (n = 3). Guidelines used by this study included RTOG, ESTRO Advisory Committee for Radiation Oncology Practice (ACROP), and Faculty of Radiation Oncology Genito-Urinary Group (FROGG), where 8 studies did not specify guidelines. Only 3 prostate studies were performed by more than one institution, and only one study reported DLAS of prostate regional nodes. CT scans (n = 8) and in-house models were used for treatment planning and DLAS contouring (n = 8) more often than MRI scans (n = 4) and commercial models (n = 4). DSC (n = 10), HD (n = 6), and qualitative or subjective evaluation methods (n = 6) were used most often to assess model performance.

Overall, the DSCs for CTV prostate ranged from 0.65 to 0.92.^56^^,^^58^, ^59^, ^60^, ^61^, ^62^, ^63^, ^64^, ^65^, ^66^, ^67^ Most DLAS models had DSC >0.7, with the exception of U-Net in one study.^64^ In intact patients with prostate cancer, the use of DLAS models demonstrated superiority, as blind physician evaluation resulted in selection of DLAS more often than manual contouring.^65^ For patients who received radiation after prostatectomy, DLAS models either outperformed or performed similarly to manual contouring.^60^^,^^63^ However, one study showed DLAS-generated CTVs were scored acceptable in 54% of the cases after prostatectomy, compared 73% for manual delineations.^62^ Models which allowed for adaptability to physician style had an average DSC 3.4% higher than with a general model which did not differentiate physician style.^60^ DLAS model performance on CT scans versus MRI scans was comparable with median DSC values of 0.84 (R, 0.7-0.88) and 0.855 (R, 0.65-0.92), respectively. Also, commercial versus in-house model performance was similar with median DSC values of 0.83 (R, 0.7-0.88) and 0.855 (R, 0.65-0.92), respectively. When evaluating pelvic lymph nodes in prostate cancer, Rayn et al reported few or minimal edits required for 99% of DLAS lymph node contours.^43^ Few articles (n = 1), reported on time savings for prostate contouring, with Shen et al showing an average contouring time of <15 seconds.^65^ From our review, one article reported dosimetric data for CTV, which showed agreement among the DLAS model and manual contours in regards to D_98%_, D_2%_, and V_95%_.^66^ The use of DLAS shows potential for increased accuracy and efficiency of CTV contours for both intact and postprostatectomy patients with prostate cancer.

## Discussion

The study of DLAS of CTV has increased in the past 4 years, especially in disease sites such as the cervix and prostate. This could be due to the high contouring time it takes for external beam cervical cancer cases and high prevalence of prostate cancer. Even more common cancers, such as lung cancer, did not have DLAS studies meeting our review criteria. DLAS models show promise of accurate contouring of CTV volumes for multiple disease sites based on reported DSC and HD values. Most DLAS models perform CTV contouring faster than manual contouring. In the few articles that reported dosimetric performance, namely in breast and cervical cancer, DLAS models did not perform as well as ground truth contours. These models could reduce the workload burden on radiation oncologists, as there is comparable contouring performance to manual contours and atlas-based contours. Manual contours can take up to 60 minutes, and DLAS models can often contour CTV volumes and other volumes in under 10 minutes. However, users must recognize that manual edits to DLAS contours may be required, especially to achieve optimal dosimetry. DLAS model performance may also be limited by variations of clinical guidelines used which may not be consistent with practice pattern of individual physicians or their practices, especially for CTV volumes like regional breast lymph nodes. Limitations of this review include the lack of uniformity on DLAS model performance making more advanced statistics difficult to perform, only one research database was used for literature review, and publication bias with published studies mostly showing benefit of DLAS models.

Further studies investigating DLAS of CTV volumes are necessary, and there are several improvements that can be made. Future DLAS models investigating disease sites like prostate and rectal cancer require extensive studies and validation in both preoperative and postoperative settings before widespread clinical implementation. Most studies have small sample sizes for DLAS model testing, are limited to data and validation at a single institution, and do not report dosimetric data. Future studies can consider using alternatives to CT imaging, such as MRI or PSMA-PET to potentially improve accuracy of DLAS models. Also, future studies should include larger sample sizes of patients from multiple institutions including dosimetric outcomes from DLAS contours to allow for more generalizable data, which may have wider clinical applicability. Larger sample sizes of patients should include breakdown of model performance according to stage of cancer and patient demographics such as race and sex assigned at birth to better characterize model performance and adaptation to real-world patients. Furthermore, explicit description of guidelines should be enforced across disease sites to allow for consistency.

## Conclusion

DLAS will bring significant improvement to the future of contouring within the field, but in the interim, more studies must be done to account for the limitations in data present.

## Disclosures

Hefei Liu reports past temporary employment at Varian Medical Systems. Sushil Beriwal has a leadership role as the Vice President of Medical Affairs at Varian Medical Systems, reports grant as an Elsevier consultant, and reports participation in advisory board at Xoft DSMB.
